# Changing the paradigm for hospital outbreak detection by leading with genomic surveillance of nosocomial pathogens

**DOI:** 10.1099/mic.0.000700

**Published:** 2018-07-27

**Authors:** Sharon J. Peacock, Julian Parkhill, Nicholas M. Brown

**Affiliations:** 1 London School of Hygiene and Tropical Medicine, Keppel Street, London WC1E 7HT, UK; 2 Department of Medicine, University of Cambridge, Addenbrooke’s Hospital, Box 157 Hills Road, Cambridge CB2 0QQ, UK; 3 Wellcome Sanger Institute, Hinxton, Cambridgeshire CB10 1SA, UK; 4 Clinical Microbiology & Public Health Laboratory, Cambridge University Hospitals NHS Foundation Trust, Addenbrooke’s Hospital, Box 236 Hills Road, Cambridge CB2 0QW, UK

**Keywords:** bacterial sequencing, transmission, outbreak detection, nosocomial

## Abstract

The current paradigm for hospital outbreak detection and investigation is based on methodology first developed over 150 years ago. Daily surveillance to detect patients positive for pathogens of particular importance for nosocomial infection is supported by epidemiological investigation to determine their relationship in time and place, and to identify any other factor that could link them. The antibiotic resistance pattern is commonly used as a surrogate for bacterial relatedness, although this lacks sensitivity and specificity. Typing may be used to define bacterial relatedness, although routine methods lack sufficient discriminatory power to distinguish relatedness beyond the level of bacterial clones. Ultimately, the identification of an outbreak remains a predominately subjective process reliant on the intuition of experienced infection control professionals. Here, we propose a redesign of hospital outbreak detection and investigation in which bacterial species associated with nosocomial transmission and infection undergo routine prospective whole-genome sequencing. Further investigation is based on the probability that isolates are associated with an outbreak, which is based on the degree of genetic relatedness between isolates. Evidence is provided that supports this model based on studies of MRSA (methicillin-resistant *Staphylococcus aureus*), together with the benefits of a ‘Sequence First’ approach. The feasibility of implementation is discussed, together with residual barriers that need to be overcome prior to implementation.

The current paradigm for hospital outbreak detection and investigation is based on a combination of surveillance and epidemiology, which is sometimes referred to as ‘shoe leather epidemiology’. The origins of this approach dates back more than 150 years ago to John Snow, a public health physician and one of the founders of modern epidemiology. He is best known for his work on cholera and, in particular, his investigation of a cholera outbreak in Soho in 1854 [1]. Mapping deaths from cholera, combined with a detailed investigation of the consumption of water from a pump in Broad Street, led Snow to propose that there was a direct link between the two and enabled him to convince the local council to remove the pump handle. This was particularly impressive since the prevailing theory for infectious disease causation at the time was based on miasma (bad air) rather than germ theory.

Hospital outbreak detection, investigation and control fol-lows a paradigm that is little changed from the methodology described by John Snow (Fig. 1a). Daily surveillance is undertaken for patients who are positive for pathogens of particular importance for nosocomial infection, including methicillin-resistant *Staphylococcus aureus* (MRSA), *Clostridium difficile* and Enterobacteriaceae that are resistant to the carbapenem drugs. The detection of two or more patients who are positive for the same indicator organism is investigated using hospital databases and ward visits to determine whether cases have overlapped in time and place, and to identify any other factor that could link them. All of the available information is then reviewed to assess the probability of an outbreak and the need for further investigation (e.g. screening other patients, staff and/or equipment) and interventions (e.g. enhanced infection control measures, cleaning). Some of this data analysis can be automated using currently available commercial infection control IT systems directly feeding off laboratory data, but ultimately the identification of an outbreak remains a predominately subjective process reliant on the intuition of the infection control professionals involved.

Bacterial typing to determine the degree of relatedness between isolates cultured from patients involved in a putative outbreak may be used downstream of surveillance and clinical epidemiology. Enthusiasm for bacterial typing during an outbreak investigation is tempered by the failure of commonly available typing methods to provide information that influences decisions or practice. Typing is often performed in centralized reference laboratories and results may take days or weeks to reach the sender because of the accumulated time taken to transport and process the isolate, by which time the need to establish relatedness has often dwindled. Available methods also fail to distinguish between isolates that belong to the same clone. This is clinically important because many nosocomial pathogens often belong to a restricted number of clones. For example, around three-quarters of all MRSA associated with invasive disease or carriage in healthcare or community settings in the United Kingdom (UK) are multilocus sequence type (ST) 22 [2], which is poorly resolved by other typing methods such as pulsed-field gel electrophoresis or *spa* typing. As a result, establishing that all isolates from a patient cluster are ST 22 neither confirms nor refutes an outbreak.

The first indication that bacterial whole-genome sequencing could have major utility for outbreak investigation came in 2010 with the publication of a study that compared the genomes of 63 MRSA isolates drawn from a global collection isolated between 1982 and 2003, all of which belonged to a single clone (ST 239) [3]. The phylogenetic analysis was based on variable genetic sites in the core genome (genes that are conserved across the species). This demonstrated extensive genetic heterogeneity, with a total of 4310 variable sites detected across the collection and no two strains were identical. Comparison with the results of pulsed-field gel electrophoresis and *spa* typing confirmed the overwhelming superiority of sequencing based on degree of discrimination between isolates. Global structuring was identified based on phylogenetic clustering of genomes by geographic region of isolation, and there was genetic evidence for intercontinental transmission. Of particular note for hospital-based infection control was the observation that 5 of 20 isolates collected over 7 months at a single hospital in Thailand were differentiated by only 14 single nucleotide polymorphisms (SNPs), which, based on the estimated rate of evolution over time (one core genome SNP every 6 weeks), indicated that they were highly related. Four of these isolates had been cultured within a 16-day period from patients located in adjacent hospital blocks.

Although the ability to sequence and compare numerous bacterial genomes represented a major technological advance, one of the limitations for its translation into infection control practice was the time taken to generate sequence data. The global MRSA collection described above was sequenced using an Illumina Genome Analyzer GAII [3], which was designed with human genomes in mind and generated large amounts of sequence data over a run time of more than a week. This meant that sequencing of much smaller bacterial genomes using this instrument was inefficient and costly unless large numbers of isolates were sequenced together (multiplexed), and was too slow for use in an acute outbreak. This barrier was overcome with the development of benchtop instruments such as the Illumina MiSeq, which at the time of release was capable of sequencing around 10 isolates at the same time with a run time of around a day. The first application of the Illumina HiSeq to an outbreak investigation was published in 2012 [4]. This proof-of-principle study for sequencing of hospital outbreaks undertook a retrospective investigation of an MRSA ST 22 outbreak in a neonatal intensive care unit, and demonstrated that isolates (patients) involved in an outbreak could be separated from those that were not. This study also presented early evidence that sequence data could be used to accurately predict phenotypic antibiotic susceptibility, and to detect the presence of numerous toxin genes.

The next step was to determine the added value of bacterial sequencing versus what could be achieved through standard infection control investigation alone. A study published in 2013 demonstrated not only significant superiority of standard practice plus sequencing, but also that this could bring about an infection control intervention that was associated with cessation of an outbreak [5]. The study began as a retrospective investigation of a cluster of MRSA cases in a special care baby unit (SCBU), where weekly MRSA screening of all infants was part of routine practice. The identification of three MRSA-positive infants in the SCBU with overlapping admission dates led to a major infection control investigation, including a 6-month look-back exercise. Seventeen MRSA-positive infants were identified in this 6-month period. After a detailed review, these cases were not considered to represent an extended outbreak, in part because there were several gaps in time when no MRSA cases were identified by screening. By contrast, sequencing identified that 14 infants were positive for MRSA that were highly related, providing strong evidence for an outbreak. Armed with this information and the subsequent detection of a new case who carried the outbreak strain, an investigation was conducted of MRSA carriage by staff. Rapid sequencing of MRSA isolated from a staff carrier using a benchtop instrument confirmed that they carried the outbreak strain. The staff member was temporarily removed from the ward and decolonized, which was associated with a cessation of the outbreak.

The SCBU study also identified the potential for sequencing to discover unsuspected nosocomial MRSA transmission [5]. Additional sequencing of 19 MRSA isolates cultured in the routine laboratory and that were chosen without prior epidemiological information (but selected on antibiotic resistance pattern) identified that the outbreak strain had spread into the community, and that these isolates were associated with clinical disease. Epidemiological investigation identified that the people affected were infants who had been inpatients on SCBU but were not known to be MRSA positive by the time of discharge: mothers of infants, some of whom were not SCBU inpatients (suggesting spread between mothers in the maternity ward); and partners of affected mothers and/or infants (Fig. 2) [5]. This degree of forensic epidemiology was only possible because of the genomic information, which allowed the tracing of individual transmission pathways within and between families. A follow-on study demonstrated that the SCBU outbreak strain had persisted in the community population since its introduction [6].

Sequencing has also been shown to have utility in ruling out outbreaks in instances where patient clusters have occurred by chance [7]. Sequencing of MRSA associated with five episodes of bloodstream infection in four patients who had overlapping admissions to a specialist hepatology unit demonstrated unequivocally that the four cases were unrelated [7]. Placing the bloodstream isolates within a local and global phylogenetic tree of MRSA genomes from the same lineage (ST 22) demonstrated that the isolates from the four patients were highly diverse (Fig. 3). This was consistent with the acquisition and importation of each MRSA from the wider referral network. Refuting outbreaks could reduce unnecessary infection control investigation and interventions. Furthermore, no sequence data are wasted since these provide the potential pool from which new outbreaks could arise, and so represent important genetic context for prospective sequencing and genomic comparisons.

Bacterial sequencing has also been used as a research tool to investigate nosocomial outbreaks caused by a range of additional bacterial species, including *Clostridium difficile* and multidrug-resistant Gram-negative bacteria such as *Klebsiella pneumoniae* [8–11]. Although not the focus of this review, sequencing has also been evaluated and introduced for the detection of foodborne outbreaks, and to predict phenotypic antibiotic susceptibility and transmission of *Mycobacterium tuberculosis* [12–14]. These studies provide unequivocal evidence for the superiority of sequencing to distinguish whether a range of bacterial species are linked to an outbreak.

Having established that bacterial sequencing could introduce a major enhancement to infection control practice, a key question is whether this should be used as a late adjunct when an outbreak is suspected or under way (reflecting the way that typing is currently implemented), or used more proactively as proposed in Fig. 1(b). Sequencing and realtime analysis of all isolates belonging to specific bacterial species as a matter of routine could provide an early warning system and provide the opportunity to intervene and prevent further transmission and involvement of new cases. However, this would represent a fundamental paradigm shift in practice, with sequence data taking a leading role in directing outbreak investigation activity. Supporting its proactive use is the observation in the SBCU outbreak study described above [5] that sequencing of MRSA isolates from the first two infants involved in the outbreak could have resulted in much earlier detection and control, and may have prevented the associated morbidity from infection in later cases. A shift in practice of this magnitude, however, requires evidence that goes beyond observation.

The first published evidence for the utility of proactive sequencing came from a study of genomic surveillance of MRSA isolated in a large clinical microbiology laboratory in the East of England over 12 months [15]. This laboratory received samples from 75 General Practitioner (GP) surgeries and 3 hospitals, and during the study period identified 1465 people who were carrying and/or infected by MRSA, from whom 2282 MRSA isolates were sequenced [15]. Around 80% of isolates were from samples submitted by hospitals, and the remaining samples originated from GP surgeries. Integration of genomic and epidemiological data led to the identification of 173 separate transmission clusters containing between 2 and 44 cases and involving 598 people, none of which were detected by conventional infection control approaches. Furthermore, 27 clusters occurred outside of hospital, one of which involved 15 people linked to a single GP practice, the majority of whom had attended a leg ulcer/podiatry clinic [16]. From this, it is clear that proactive sequencing detects many more outbreaks, and identifies linkages between hospitals and the community.

There has been considerable debate in the past about where pathogen sequencing should be performed, and in particular whether this should be centralized (for example, using facilities developed for human genome sequencing), or distributed throughout the network of hospital-based diagnostic laboratories. The need to generate and interpret data in the shortest possible time so that ongoing transmission can be prevented means that this is most likely to be supported by local sequencing in large diagnostic laboratories. Hospitals that maintain smaller laboratories that cannot justify the cost of implementation could refer isolates to reference laboratories, or alternatively use commercial sequencing providers although the turnaround time could prove a limitation [17]. However, the pace at which sequencing instruments are being developed for clinical practice means that this technique could be done anywhere with minimum training in the near future, rendering this debate obsolete. If bacterial sequencing is to be introduced locally, this will need to be technically feasible but is largely already within the capabilities of larger diagnostic laboratories. Methods for DNA extraction are in widespread use, preparation of DNA sequencing libraries is no more complex than many other diagnostic molecular methods, and commercially available sequencing instruments are simple to use. Furthermore, a laboratory with a high throughput of bacterial sequencing could justify the additional capital costs associated with automation of DNA extraction and library preparation. Standardized protocols will be required, but their development and dissemination could follow current best practice for the adoption of any laboratory method.

Sequence data will need to be generated in a timescale that provides actionable information, guiding and improving practice rather than providing a retrospective view of no clinical impact or benefit. This is also achievable. The shortest possible turnaround would be achieved by sequencing bacteria directly from the patient specimen, but this is largely beyond what is technically feasible at the present time. Sequencing is currently performed from DNA extracted from a pure bacterial culture, which introduces a delay of around a day. This can be circumvented by performing DNA extraction and library preparation directly from bacterial colonies growing in the primary culture [18]. This has been described for 17 bacterial pathogens responsible for severe human disease that were grown using standard diagnostic media and incubation conditions. Colony pick to completion of DNA library preparation can be achieved in around 4 h using manual methods. The time taken to generate sequence data will depend on the sequencing instrument used, which for laboratory benchtop instruments ranges from several hours to a day. Important considerations when deciding on the appropriate sequencing instrument are cost (equipment, kits and reagents), throughput (matched against how many isolates a laboratory aims to sequence per day), time (for preparation and sequencing) and accuracy. Accuracy of sequence data is particularly important for outbreak investigations where the number of core genome SNPs is used to define relatedness. Accuracy will also be important for other indications, including the genetic prediction of resistance to an antibiotic that is mediated by point mutations in a chromosomal gene.

Whilst most barriers to the clinical use of genomic surveillance have now been overcome, the most important remaining hurdle is the automated analysis of sequence data. Although the analysis of bacterial genomes is facilitated by the availability of numerous publicly available software scripts, this is highly specialized, time consuming and does not result in a read-out that is clinically meaningful to the majority of infection control staff. Several research groups and commercial companies are actively developing tools that automate the interpretation of bacterial sequence data specifically for use by infection control teams, and it is likely that tools will become available for the analysis of several of the major nosocomial pathogens in the next 12–18 months. Data on potential outbreaks could be fed back to infection control teams in real time to allow epidemiological information to be added and the outbreak confirmed or refuted. This is an important step, since genomic and epidemiological information are complementary. The epidemiological information need not be onerous to collect and is likely to be readily available on hospital systems. While the most useful information might vary from one organism to another, for MRSA it was identified that recent hospital admissions, hospital ward, home postcode and registered GP practice were most helpful [15]. The report generated for infection control teams needs to be fully comprehensible to users without bioinformatics training. A proposed whole-genome sequencing clinical report has been devised for *M. tuberculosis* using a process of evidence-based design and evaluation [19], much of which is relevant to outbreaks in hospitals caused by other pathogens.

Software that determines bacterial genome relatedness for outbreak investigation should be able to compare genomes generated within and between sequencing runs. Individual laboratories will want to accumulate their own reference library of genomes so that the latest data can be compared with those sequenced in the preceding days, weeks or months. Shorter timescales for genomic comparisons would identity active outbreaks, while links to isolates cultured over a longer timeframe may identify cryptic transmission in the past and high-risk areas that warrant increased infection control scrutiny. The computing power required to compare numerous bacterial genomes is likely to require the use of cloud-based facilities, and automated analysis pipelines will need to conform to data protection standards and ensure patient confidentiality. A logical progression from local analysis would be to compare isolates from different laboratories to identify broader patterns of transmission between healthcare providers, as well as re-use of data for national surveillance. Future innovation includes the linkage of sequence data with bed management systems that track patient movement. The automated integration of genomic and patient movement data could generate a daily report of cases who are positive for a highly related organism, and immediately highlight new cases in a developing or established outbreak. Furthermore, machine learning methods could be applied to such datasets to predict where and when outbreaks are likely to occur.

Implementing genomic surveillance will be associated with an up-front cost, and the argument for introducing this innovation into routine practice will need to go beyond the purely scientific. Adopting routine genomic surveillance of nosocomial pathogens into the NHS and elsewhere will require evidence of cost effectiveness. Since carriage of nosocomial pathogens is asymptomatic, the benefit derived from genomic surveillance comes from preventing nosoco-mial infection that may follow a new acquisition event. Healthcare-associated infections caused by a range of pathogenic species prolong hospital stay, increase healthcare costs and lead to poorer patient outcomes. The benefits of proactive pathogen sequencing could include benefits to patients and financial savings. The latter may be associated with reduced patient stay and increased efficiency of infection control teams, although greater detection of true outbreaks may actually increase their workload. Economic evaluation of routine sequencing of nosocomial pathogens has not been reported to date, and is needed if the case for routine bacterial sequencing is to be successfully argued.

Arguments for the adoption of genomic surveillance could also draw on the obvious case of need, the benefits from which are felt by the entire patient population. Outbreak detection is highly complex, since this requires infection control teams to keep track of potential points of contact between hundreds or thousands of patients in a given healthcare facility on a daily basis, often in the absence of technological solutions that fully automate this. Superimposed on this is the rate of patient movement within NHS hospitals, which is at an all-time high. Patients with complex medical needs may be moved through several wards or specialities during their care pathway. In addition, the rising demand on NHS beds is being managed in part by repeatedly moving patients to maximize the use of a limited number of empty beds. Detecting outbreaks can also be very challenging when these are associated with transmission from one or more healthcare workers who work across several wards or healthcare areas, or who are based in a clinic from which patients may be admitted to unrelated wards.

Proactive sequencing of targeted nosocomial pathogens could provide a technological solution to this complex environment and generate actionable information, both in hospitals and the community.

Finally, genomic surveillance and the detection and control of hospital outbreaks is increasingly important at a time when the introduction of multidrug-resistant pathogens into hospitals is increasingly likely. Sequencing could play a substantial role in reducing the risk of drug-resistant infections in hospital patients, and the spread of such pathogens from hospitals into the community.

## Figures and Tables

**Fig. 1:**
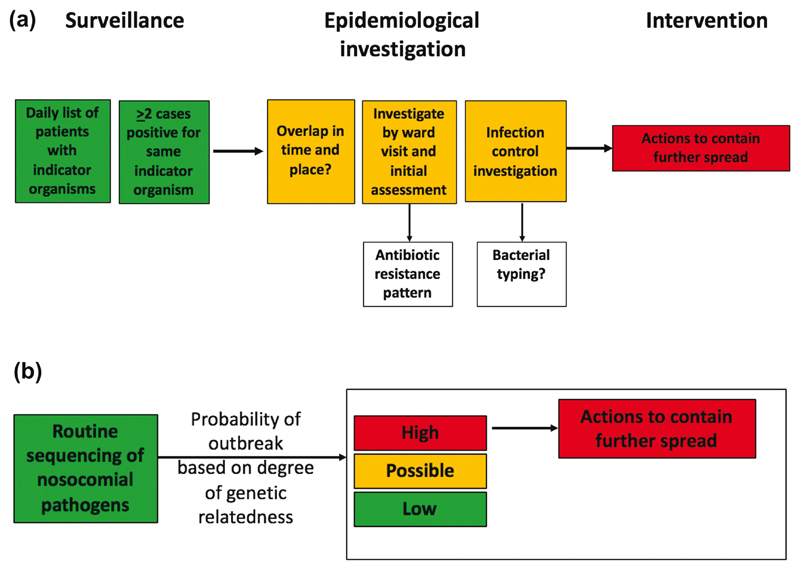
Current and proposed approach to the detection of hospital outbreaks. (a) Current practice for the detection of hospital outbreaks based on surveillance and epidemiology. The pattern of antibiotic resistance is commonly used as a surrogate for bacterial relatedness, and formal bacterial typing may be used during outbreak investigation. (b) A proposed alternative in which outbreak detection is led by routine sequencing of bacterial species that are commonly associated with nosocomial outbreaks and infection.

**Fig. 2:**
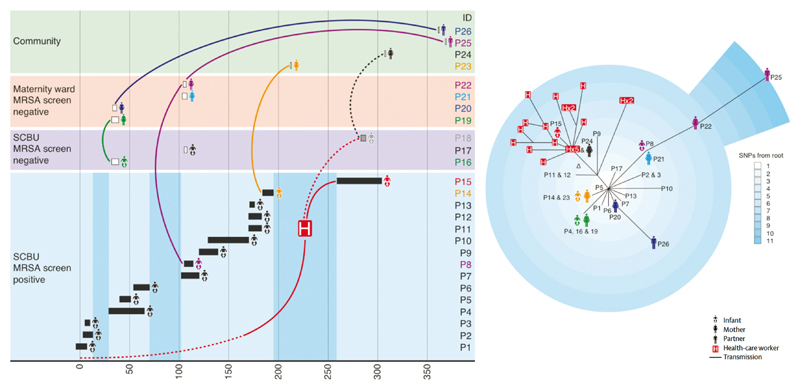
Epidemiology and phylogeny of an MRSA outbreak. Left: timeline (in days) of an outbreak that affected infants on a special care baby unit (SCBU), and that went on to affect family clusters. A total of 26 people were affected: infants treated on the SCBU who were known to be MRSA positive during admission (P1–14) or who were not known to be carriers during admission but were detected after discharge (P16-18); mothers who were (P19–22), or were not (P23–24), inpatients on the maternity ward; and partners (P25–26). The length of the boxes shown for infants on SCBU represent duration of hospital stay. A healthcare worker was detected who was also carrying the outbreak strain (denoted by H). Darker vertical blue blocks show times on the SCBU when there were no known carriers of MRSA. Right: phylogenetic tree of MRSA isolated from patients 1–26, together with 20 individual MRSA colonies from a staff member (denoted by H). SNP, single nucleotide polymorphism. Adapted from reference [5].

**Fig. 3:**
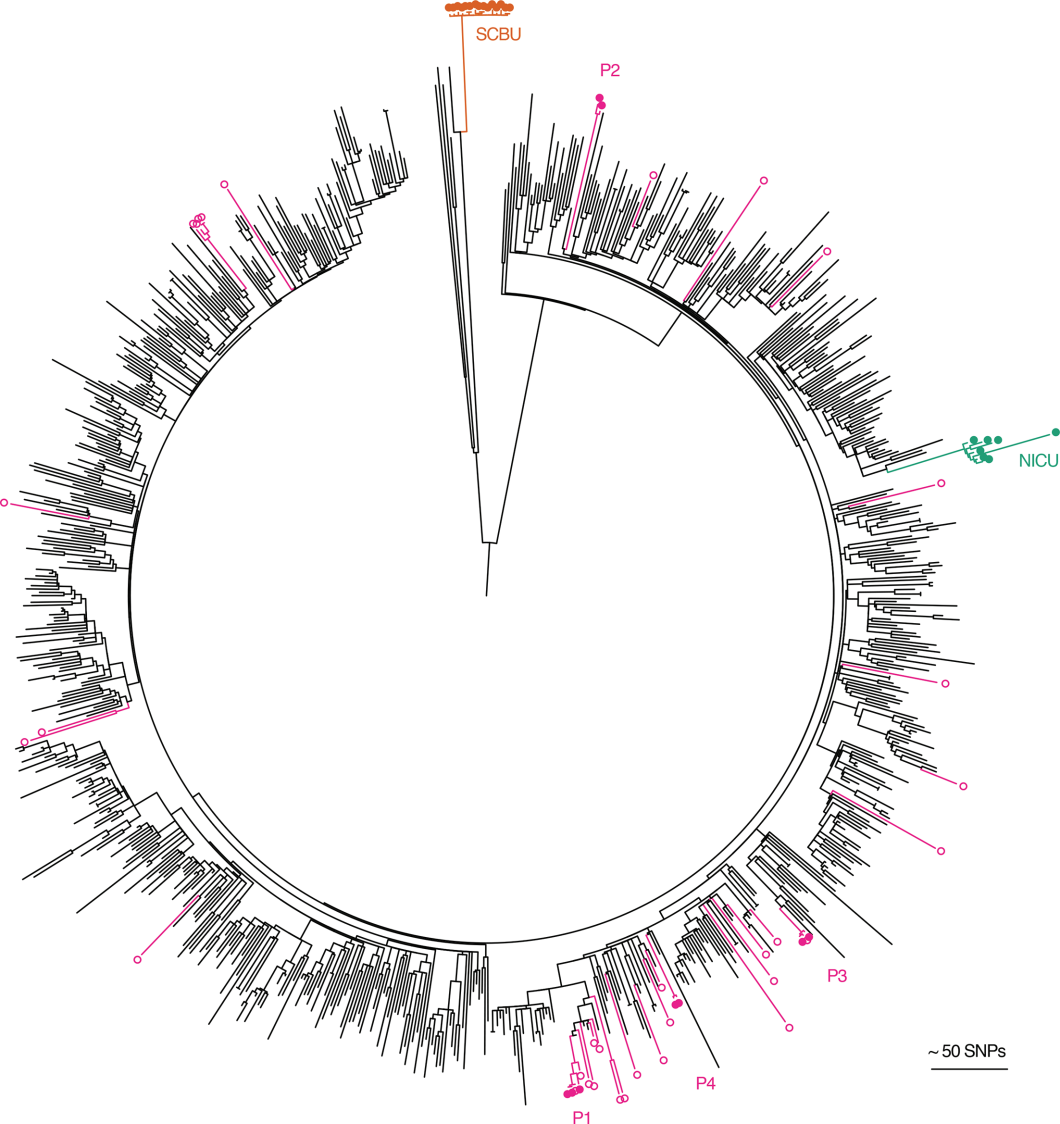
Contextualization of outbreak investigations of CC22 MRSA studied at Cambridge University Hospitals. The maximum likelihood tree was based on 22 238 core SNPs for 783 ST22 genomes drawn from the British Society of Antimicrobial Chemotherapy bacteraemia resistance surveillance programme between 2001 and 2010; 7 isolates from an MRSA outbreak on a neonatal intensive care unit (NICU, green) [4]; 15 isolates from an MRSA outbreak that focused on a special care baby unit (SCBU, orange) but extended to other wards and the community [5]; and 42 isolates sequenced as part of an MRSA outbreak investigation on a hepatology ward (nine isolates from four patients with bacteraemia (P1–4; pink filled dots); and the remainder from patients who were MRSA carriers on the same ward during a comparable timeframe (pink open dots) [7]. Reproduced from reference [2].

## Five reasons to publish your next article with a Microbiology Society journal

1. The Microbiology Society is a not-for-profit organization.
2. We offer fast and rigorous peer review - average time to first decision is 4-6 weeks.
3. Our journals have a global readership with subscriptions held in research institutions around the world.
4. 80% of our authors rate our submission process as ‘excellent’ or ‘very good’.
5. Your article will be published on an interactive journal platform with advanced metrics.

**Find out more and submit your article at microbiologyresearch.org.**

## Abbreviations

DNA: deoxyribonucleic acid
GP: General Practitioner
IT: information technology
MRSA: methicillin-resistant *Staphylococcus aureus*
NHS: National Health Service
P: patient
SCBU: special care baby unit
SNP: single nucleotide polymorphism
ST: sequence type
UK: United Kingdom

## References

[1] Snow J (1855). On the Mode of Communication of Cholera.

[2] Reuter S, Török ME, Holden MT, Reynolds R, Raven KE (2016). Building a genomic framework for prospective MRSA surveillance in the United Kingdom and the Republic of Ireland. Genome Res.

[3] Harris SR, Feil EJ, Holden MT, Quail MA, Nickerson EK (2010). Evolution of MRSA during hospital transmission and intercontinental spread. Science.

[4] Köser CU, Holden MT, Ellington MJ, Cartwright EJ, Brown NM (2012). Rapid whole-genome sequencing for investigation of a neonatal MRSA outbreak. N Engl J Med.

[5] Harris SR, Cartwright EJ, Török ME, Holden MT, Brown NM (2013). Whole-genome sequencing for analysis of an outbreak of meticillin-resistant *Staphylococcus aureus*: a descriptive study. Lancet Infect Dis.

[6] Toleman MS, Reuter S, Coll F, Harrison EM, Peacock SJ (2016). Local persistence of novel MRSA lineage after hospital ward outbreak, Cambridge, UK, 2011-2013. Emerg Infect Dis.

[7] Török ME, Harris SR, Cartwright EJ, Raven KE, Brown NM (2014). Zero tolerance for healthcare-associated MRSA bacteraemia: is it realistic?. J Antimicrob Chemother.

[8] Eyre DW, Cule ML, Wilson DJ, Griffiths D, Vaughan A (2013). Diverse sources of *C. difficile* infection identified on whole-genome sequencing. N Engl J Med.

[9] Snitkin ES, Won S, Pirani A, Lapp Z, Weinstein RA (2017). Integrated genomic and interfacility patient-transfer data reveal the transmission pathways of multidrug-resistant *Klebsiella pneumoniae* in a regional outbreak. Sci Transl Med.

[10] Gorrie CL, Mirceta M, Wick RR, Edwards DJ, Thomson NR (2017). Gastrointestinal Carriage is a major reservoir of *Klebsiella pneumoniae* infection in intensive care patients. Clin Infect Dis.

[11] Reuter S, Ellington MJ, Cartwright EJ, Köser CU, Török ME (2013). Rapid bacterial whole-genome sequencing to enhance diagnostic and public health microbiology. JAMA Intern Med.

[12] Gardy JL, Johnston JC, Ho Sui SJ, Cook VJ, Shah L (2011). Whole-genome sequencing and social-network analysis of a tuberculosis outbreak. N Engl J Med.

[13] Walker TM, Kohl TA, Omar SV, Hedge J, Del Ojo Elias C (2015). Whole-genome sequencing for prediction of *Mycobacterium tuberculosis* drug susceptibility and resistance: a retrospective cohort study. Lancet Infect Dis.

[14] Pankhurst LJ, Del Ojo Elias C, Votintseva AA, Walker TM, Cole K (2016). Rapid, comprehensive, and affordable mycobacterial diagnosis with whole-genome sequencing: a prospective study. Lancet Respir Med.

[15] Coll F, Harrison EM, Toleman MS, Reuter S, Raven KE (2017). Longitudinal genomic surveillance of MRSA in the UK reveals transmission patterns in hospitals and the community. Sci Transl Med.

[16] Toleman MS, Watkins ER, Williams T, Blane B, Sadler B (2017). Investigation of a cluster of sequence type 22 methicillin-resistant *Staphylococcus aureus* Transmission in a Community Setting. Clin Infect Dis.

[17] Raven K, Blane B, Churcher C, Parkhill J, Peacock SJ (2018). Are commercial providers a viable option for clinical bacterial sequencing?. Microb Genom.

[18] Köser CU, Fraser LJ, Ioannou A, Becq J, Ellington MJ (2014). Rapid single-colony whole-genome sequencing of bacterial pathogens. J Antimicrob Chemother.

[19] Crisan A, McKee G, Munzner T, Gardy JL (2018). Evidence-based design and evaluation of a whole genome sequencing clinical report for the reference microbiology laboratory. Peer J.

